# Supplementary material for: Seasonal Dynamics of Culturable Yeasts in Ornithogenically Influenced Soils in a Temperate Forest and Evaluation of Extracellular Enzyme Secretion in Tausonia pullulans at Different Temperatures
Source: J Fungi (Basel). 2024 Jul 30;10(8):532. doi: 10.3390/jof10080532 (PMC11355323; doi:10.3390/jof10080532)
Supplement: Supplementary file 1 [file jof-10-00532-s001.zip › jof-3081798-supplementary.pdf]

Table S1. Secretion ability of extracellular enzymes among the soil strains of the yeast *Tausonia pullulans* at different temperatures in vitro.

Strains	Esterase					Lipase					Protease				
	2°C	4°C	10°C	15°C	20°C	2°C	4°C	10°C	15°C	20°C	2°C	4°C	10°C	15°C	20°C
23bd-3	*++	++	+	+	w	+	+	+	+	w	w	w	w-	w-	−
23bd-4	++	++	+	+	w	+	+	+	+	w	w	w	w-	w-	−
23LH-2	++	++	+	+	w	+	+	+	+	w	w	w	w-	w-	−
23LH2-37	++	++	+	+	w	+	+	+	+	w	w	w	w-	w-	−
23LH2-38	++	++	+	+	w	+	+	+	+	w	w	w	w-	w-	−
23LH-10	++	++	+	+	w	+	+	+	w	w	w	w	w-	w-	−
23LH-14	++	++	+	+	w	+	+	+	+	w	w	w	w-	w-	−
23LH-19	++	++	+	+	w	+	+	+	+	w	w	w	w-	w-	−
23LH-27	++	++	+	+	w	+	+	+	w	w	w	w	w-	w-	−
23rew-3	++	++	+	+	w	+	+	+	+	w	w	w	w-	w-	−
23rew-4	++	++	+	+	w	+	+	+	+	w	w	w	w-	w-	−
23Fe-3	++	++	+	+	w	+	+	+	+	w	w	w	w-	w-	−
24mb-6	++	++	+	+	w	+	+	+	+	w	w	w	w-	w-	−
24mb-11	++	++	+	+	w	+	+	+	+	w	w	w	w-	w-	−
24mb-9	++	++	+	+	w	+	+	+	+	w	w-	w	w-	w-	−
24b-14	++	++	+	+	w	+	+	+	+	w	w	w	w-	w-	−
24b-8	++	++	+	+	w	+	+	+	+	w	w	w	w-	w-	−
24b-11	++	++	+	+	w	+	+	+	w	w	w	w	w-	w-	−
24b-1	++	++	+	+	w	+	+	+	+	w	w	w	w-	w-	−
24mb-7	++	++	+	+	w	+	+	+	+	w	w	w	w-	w-	−
24mb-8	++	++	+	+	w	+	+	+	+	w	w	w	w-	w-	−
24mb-4	++	++	+	+	w	+	+	+	w	w	w	w	w-	w-	−
24b-3	++	++	+	+	w	+	+	+	+	w	w	w	w-	w-	−
24mb-1	++	++	+	+	w	+	+	+	+	w	w	w	w-	w-	−
24b-10	++	++	+	+	w	+	+	+	+	w	w	w	w-	w-	−
24ba-6	++	++	+	+	w	+	+	+	+	w	w	w	w-	w-	−
24ap-6	++	++	+	+	w	+	+	+	+	w	w-	w	w-	w-	−
24ap-2	++	++	++	+	w	+	+	+	w	w	w	w	w-	w-	−
24ba-1	++	++	+	+	w	+	+	+	+	w	w	w	w-	w-	−
24ba-2	++	++	+	+	w	+	+	+	+	w	w	w	w-	w-	−
24ba-3	++	++	+	+	w	+	+	+	w	w	w	w	w-	w-	−
24ba-5	++	++	+	+	w	+	+	+	+	w	w-	w	w-	w-	−

24ba-4	++	++	+	+	w	+	+	+	+	w	w-	w	w-	w-	−
24bslk-3	++	++	+	+	w	+	+	+	+	w	w	w	w-	w-	−
24bslk-4	++	++	+	+	w	+	+	+	+	w	w	w	w-	w-	−
24bslk-9	++	++	+	+	w	+	+	+	+	w	w	w	w-	w-	−
24bslk-12	++	++	+	+	w	+	+	+	+	w	w	w	w-	w-	−
24fp-7	+	+	+	w	−	+	+	w	w	−	−	−	−	−	−
24bslk-13	++	++	+	+	w	+	+	+	+	w	w	w	w-	w-	−
24fp-10	++	++	+	+	w	+	+	+	w	w	w-	w	w-	w-	−
24fp2-10	+	+	+	+	w	+	+	+	+	w	w	w	w-	w-	−
24fp2-7	++	++	+	+	w	+	+	+	+	w	w	w	w-	w-	−
24bslk2-12	++	++	+	+	w	+	+	+	+	w	w	w	w-	w-	−
24bslk2-9	++	++	+	+	w	+	+	+	w	w	w	w	w-	w-	−
24bslk2-4	++	++	+	+	w	+	+	+	+	w	w	w	w-	w-	−
24ap2-2	++	++	+	+	w	+	+	+	w	w	w	w	w-	w-	−
24ap2-4	++	++	+	+	w	+	+	+	+	w	w	w	w-	w-	−
24mb2-1	++	++	+	+	w	+	+	+	+	w	w	w	w-	w-	−
24b2-1	++	++	+	+	w	+	+	+	+	w	w	w	w-	w-	−
24Fe2-3	++	++	+	+	w	+	+	+	w	w	w-	w	w-	w-	−
24bd2-3	++	++	+	+	w	+	+	+	+	w	w	w	w-	w-	−
23bd2-4	++	++	++	+	w	+	+	+	+	w	w-	w	w-	w-	−

*Secretion of hydrolytic enzymes: ++ strongly positive, + positive, w weak positive, w- weak negative, – negative.
